# Weighting for Health: Management, Measurement and Self-surveillance in the Modern Household

**DOI:** 10.1093/shm/hkw015

**Published:** 2016-05-07

**Authors:** Roberta Bivins, Hilary Marland

**Keywords:** weight, health, household, measurement, self-surveillance

## Abstract

Histories of late nineteenth- and early twentieth-century medicine emphasise the rise of professional and scientific authority, and suggest a decline in domestic health initiatives. Exploring the example of weight management in Britain, we argue that domestic agency persisted and that new regimes of measurement and weighing were adapted to personal and familial preferences as they entered the household. Drawing on print sources and objects ranging from prescriptive literature to postcards and ‘personal weighing machines’, the article examines changing practices of self-management as cultural norms initially dictated by ideals of body shape and function gradually incorporated quantified targets. In the twentieth century, the domestic management of health—like the medical management of illness—was increasingly technologised and re-focused on quantitative indicators of ‘normal’ or ‘pathological’ embodiment. We ask: in relation to weight, how did quantification permeate the household, and what did this domestication of bodily surveillance mean to lay users?

Historians have depicted the nineteenth and twentieth centuries as typified by the increased dominance of professional medical authority and the rise of scientific medicine.^1^ In contrast, we argue that the household remained a primary site of health-related decision making and consumption, while incorporating both medical techniques and methods of self-surveillance. Taking one case study—the management and measurement of weight—our article explores nineteenth- and twentieth-century persistence, and even expansion, of domestic agency and activity in the pursuit of health. We examine the myriad new forms of information, techniques and devices created and marketed specifically for household use as a way to access householders themselves, who often remain silent in the historical record. Together with the more fragmentary direct evidence available about users and choosers in the home, these sources generate a picture of British homes as vibrant sites of health agency and medical decision taking. Such close scrutiny illuminates the diverse ways in which nineteenth- and twentieth-century householders acquired and responded to information on the regulation of food intake and the relationship between weight, health and physical appearance. Focusing on the under-studied British context (but informed by the rich literature on US weight management), our analysis interrogates the sources of information available to householders on weight and its management.^2^ We ask how and when the shift from surveying weight in terms of visual appearance and bodily function to assessing it via actual measurement occurred; and what were the effects of this shift on the household as a site of health management? Did the advent of particular models of surveillance—in particular, the gradual rise of domestic technologies of exact measurement—have any impact on domestic agency? Were patients, consumers and health seekers *empowered* by increased access to the tools of professional medicine (here, advice on diet, dietary tables and scales), or *colonised* by them?

Our article commences with a discussion of the contribution of household guides to health in offering advice and guidance on the management of weight in the home. We explore how mechanistic approaches of self-surveillance were absorbed into a literature that already extolled moderate food intake, encouraged householders to take responsibility for weight management, and instructed them in the skills of exact measurement. After briefly introducing the rise and especially the commercial diffusion of quantitative approaches to health—and with them, the emergence of normative responses to weight variation—we turn to the role of the adult personal scales in particular. While the impacts of this health technology (and indeed the increasing emphasis on professional and lay approaches to weight management) in the United States have attracted some scholarly attention, little has yet been written about the different trajectory and meanings of the ‘personal weighing machine’ in Britain.

## Health Guides and Weight Monitoring in the Nineteenth-century Home

Excessive weight has long attracted medical and lay attention; notable figures of vast girth and bulk generated public fascination, derision, mirth and admiration while their expanding waistlines were described and illustrated in paintings, novels, pamphlets and the press. By the end of the eighteenth century, routine corpulence had become a source of medical, literary and popular concern, exemplified by physician George Cheyne’s closely recorded and miserable battle with weight gain and equally impressive weight loss.^3^ In his campaign to shed excess pounds Cheyne took the waters at Bristol and Bath, and spa treatments became popular among well-to-do patients eager to lose weight and improve bodily tone. By the mid-nineteenth century, hydropathy, with its rigorous regimes of cold water treatments, pummelling and massage, temperate diet and open-air exercise, reproved the moral failings of overindulgence while treating its physical effects. Meanwhile, dietary guides and public weighing serviced an increasing fascination with measuring weight and moderating it.^4^ The underlying idea that weakness of will caused obesity, Gilman has argued, became a medical as much as popular trope by the early nineteenth century.^5^ Thus in 1826 when Jean Anthelme Brillat-Savarin declared obesity ‘a most unpleasant state of health’, he also moralised that it was ‘one into which we almost always fall because of our own fault’.^6^

In the mid- to late nineteenth century, diet products—from crackers and healthy cereals through to patent medicine products, including anti-fat pills such as ‘Figuroids’ (promising a ‘Scientific Obesity Cure’, reducing fat cells to normal cells) and electric belts (to stimulate weight loss)—became widely available in Britain, reflecting the huge increase in the availability and promotion of health related goods.^7^ Peter Stearns has suggested that by the late nineteenth century fat in America, too, was obsessively discussed and ‘vigorously reproved’, and diet aids and devices became common. The emergence of new fat fighting products intersected with fashion for thinness, media interest in dieting, and the production of insurance tables proclaiming norms of height and weight.^8^ Yet despite such signs of a growing trans-Atlantic cultural, medical and commercial preoccupation with expanding body weight, relatively little is known about its actual management in the home, particularly in Britain. How did families acquire and act upon information about weight loss or gain and nutrition more generally? Did householders worry about their own or others’ excessive fatness or thinness? Did they call in medical assistance to deal with weight gain or tackle it themselves?

Household medical guides illustrate the ways in which diet and attention to weight were introduced to the nineteenth-century home. The growth in the number of such publications and their frequent re-publication in large print runs testifies to the strong market for such guides, as part of a wider expansion of advice literature targeted at families.^9^ Certainly, they were purchased. William Buchan’s (1729–1805) *Domestic Medicine*, first published in 1769, sold over 80,000 copies by the time of his death in 1805, with new editions appearing roughly every two years.^10^ *Domestic Medicine* remained popular during the nineteenth century, but was challenged as a brand leader by numerous other guides to health.^11^ As Charles Rosenberg has convincingly argued, the shabbiness of popular health books surviving from this period, supplemented by householders’ newspaper cuttings and the insertion of recipes, indicates that such volumes were ‘not just read; they were used’.^12^ Guides were authored by regular physicians as well as a diverse range of heterodox practitioners. Those produced by orthodox doctors acknowledged that patients—whatever the opportunities for ‘professional’ advice and intervention—would continue to treat themselves, a process driven by purse, preference and practicality. Advocates of new healing approaches, including the nineteenth-century systems of hydropathy, homoeopathy, medical botany and vegetarianism, meanwhile, infused their attempts to empower families with knowledge and technical skills with a missionary zeal, encouraging them to actively treat a variety of medical disorders and to initiate interventions to improve their general health and well-being.^13^ Such advice was adapted to particular constitutions, ages and lifestyles, and most guides incorporated lengthy sections on health maintenance and food, diet and exercise, equipping households with rich sources of information on, amongst many other health matters, the management of nutrition and weight.

Recommendations on diet were connected to instruction on regimen and ‘rules of health’ to be observed in householders’ daily practices, insisting on the necessity of paying attention to fresh air, exercise, clothing and adequate sleep.^14^ Typically they were straightforward and easy to adopt, translating middle-class ideology into ‘physiological terms’, advocating moderation, self-control and persistence.^15^ Thus, the section on diet in Thomas Graham’s *Modern Domestic Medicine* recommended a temperate approach, carefully regulated according to age, gender and activity. ‘There can be no doubt’, Graham declared, ‘that the majority of the more respectable inhabitants of Great Britain eat and drink twice as much as is beneficial’, ignoring the physiological ‘alarm’ provided by their own sated stomachs.^16^ All were discouraged from cramming themselves ‘with anything which opportunity offers to lay their hands on’.^17^ Similarly, *Gardner’s Household Medicine and Sick-Room Guide* declared that obesity ‘was usually due to the amount of food being taken in access of requirements of the body’. He blamed ‘[h]igh living’, excessive drink, and ‘want of exercise’. To reduce fat, Gardner recommended active exercise, lowered starch consumption, avoidance of sugar and wine and the substitution of bread with thin, browned toast and hard biscuits.^18^ Likewise, M’Gregor-Robertson’s 1890 *Household Physician* devoted 100 of its hefty 1,000 pages to food types, diet, energy production, calories, cooking and the digestibility of food.^19^ He presented precise information on the composition and value of different foods, a series of dietaries and calculations of the calories required according to age, employment, bodily condition, climate and season, and described the impact of deficient and excessive diets.

Health guides produced by advocates of new medical systems were equally unambiguous about the responsibility borne by individuals and householders in managing health, and concluded that gluttony produced poor health and illustrated moral weakness. Such texts offered guidance on eating as *moral* education, whereby the stomach was to be ruled by the head and regimes of moderation and regularity encouraged. Healthy living meant living naturally. The Olsens’ 1906 *School of Health* promoted hygienic practices, simple lifestyle, natural remedies, exercise and the ‘adoption of simple, natural [and vegetarian] diet’ as the ‘most powerful aid to pure living’.^20^ Followers of US health reformer Dr John Harvey Kellogg and editors of the *Good Health* journal, the Olsens, spurned dairy products and beverages such as tea and coffee, and alcohol, and advocated ‘Fletcherism’ or chewing reform as a dietary aid.^21^ Obesity, they concluded, was brought on by poor, sedentary habits and high living, and could be cured by restricted diet, drinking large quantities of water, bathing and rigorous exercise. For the Olsens—and many other writers on health—bodily size was to be restrained largely through the regulation of behaviour and emotion in response to environmental and cultural cues, rather than those supplied by the actual measurement of girth and weight.

By the late nineteenth century, health advice was becoming ever more specialised. Guides catered for discrete audiences and no more so than for women and girls, many of whom were interested in regulating body weight for reasons of beauty and fashion as much as health. Health advice handbooks, health periodicals and magazines for women and girls elaborated increasingly on the ways and means of controlling weight, improving the figure and developing correct proportions. The problems of ‘thin’ or ‘lean’ girls and ‘fat’ or ‘obese’ girls were to be resolved by intense self-surveillance and rigorous regimes of body management; being overweight in particular was a sign of poor character and greed.^22^ Thus, in 1886 Anna Kingsford, health reformer and vegetarian, produced a health guide for women, presented in the form of letters to ‘fictional’ characters taken from the pages of the *Lady’s Pictorial* where they had first appeared. She described how ‘Julia’ was instructed to abandon her suicidal consumption of bread, potatoes, milk soup and tapioca pudding, and warned not to take pills for the mitigation of obesity. Instead, she was urged to become an early riser, to take brisk walks and exercise and to consume uncooked fruits, vegetables, white fish, lemon tea and rusks while avoiding farinaceous dishes, milk, sweets, pastry, cocoa and alcohol. Kingsford also forbad lolling in bed and eating ‘stray cakes or cups of tea’ in favour of gymnastic exercise and Turkish baths.^23^ Physical culture advocate Dr Emma Walker affirmed that ‘Every pound of flesh beyond that which is necessary to make the form symmetrical is an additional weight to carry, a burden to overcome, and a hindrance to normal functions. In other words it stands ready to destroy both health and beauty.’^24^ As advice about weight control and regimen permeated British households, it is crucial to note that moral and ‘medical’ (or more properly physiological) responses to obesity remained tightly enmeshed. The widely-perceived need for weight control remained only one part of a wider agenda of domestic management of the self. Close self-scrutiny and attentive self-surveillance were essential to both sides of this equation for health maintenance.

## Enumerating the Normal

In general, nineteenth-century health guides intended for the household reader did not yet emphasise surveillance by numbers. Not until the end of the century would they include straightforward tables relating ideal weight to gender, height, physical development, occupation and exercise, and cite hospital dietaries or diet tables drawn up by physicians as useful guides to what individuals should eat.^25^ However, household medical guides routinely offered detailed instructions on the precise measurement of the components of home remedies and on dosage. They introduced householders to a wide array of measuring paraphernalia—from graduated wine glasses, funnels and measures to scales and weights—for manufacturing and dispensing remedies, encouraging the adoption of practices of exact measurement.^26^ So while householders were not actually weighing themselves, they were becoming increasingly familiar with the culture of precise measurement as an adjunct to domestic health maintenance. Though the weight of obese celebrities was recorded with interest, as were the ‘circumference and visible volumes and contours’ of arms, legs and especially waistlines, for most people, measuring for weight was a rarity in the early nineteenth century.^27^

In contrast, specific quantification became an increasingly visible aspect of professional/expert responses to excessive weight or leanness in the mid-nineteenth century. Indeed, measurement had already come to professional medicine with a vengeance in the late eighteenth and early nineteenth centuries, via the *méthode numérique* and *méthode anatomo-clinique* of the Parisian clinic. Crucially, these techniques established the idea of ‘normal’ bodies.^28^ Quantification was at the heart of these rapidly diffusing innovations, just as statistics would be at the heart of the nineteenth-century’s major public health battles. In 1830, Adolphe Quetelet, credited as the inventor of the ‘average man’, asserted the urgent necessity of establishing human norms in health, in order to gain for medicine the analytical power ‘derivable from corporeal measurements’.^29^

New technologies of exact measurement played an essential—if controversial—role in this endeavour. As surgeon, physician and medical examiner to a large insurance company, John Hutchinson (1811–1861) wrote in 1846, ‘All we know is gathered from physical observation, through the medium of the senses’.^30^ But the senses were, by their nature, idiosyncratic. Moreover, sensory data, if captured only in words could not be minutely compared between practitioners or cases. In contrast, the new measuring instruments could provide exactly the definitive information required in the form of a numerical reading, readily comparable to similar readings taken for other patients. Hutchinson lauded the advantages offered to the medical professional by all such technological aids: their use ‘requires no delicate training of the judgment’ to produce a reliable ‘fact’. Rather, such tools provided ‘ready and definite’ data upon which to build diagnoses or health assessments, ‘without a long system of education’.^31^ In other words, as Stanley Reiser noted, standardised, calibrated instruments allowed all doctors—‘whether able or inept’—to gather accurate data about their patients’ bodies.^32^

But numbers alone, no matter what they measured, were useless. The rise of quantification in clinical practice required both the organised and systematic collection of the individual measurements of numerous individuals, and the correlation of those numbers with other quanta of embodiment: height, age, occupation, appearance—and especially weight. Speaking directly to his peers in the growing insurance industries of Britain and the United States, Hutchinson extolled weight as the most reliable predictor of health: ‘The weight is an expression of the whole man—the volume of his make; a measure of his general health . . .’.^33^ His own tables, which collated spirometric data, height and weight measurements, and information about age, ‘general appearance’ and occupation for thousands of individuals, were rapidly adopted and adapted by insurance companies in both nations.^34^ Hutchinson even urged the British government to incorporate height and weight questions into the national census, as vital indicators of ‘the social and commercial welfare of the country’.^35^

While the British government ignored Hutchinson’s pleas, the insurance industry on both sides of the Atlantic rapidly adopted the quantitative methodology. Companies would later publish much of this work in health promotion materials for their own clients, thus spreading the medical model of precise measurement into the domestic sphere.^36^ Reproducing the standard of weight and height from Association of Life Insurance Medical Directors in the London periodical the *Review of Reviews* in 1909, the editors remarked that ‘overweight universally shortens life. Overweight is a burden, not a reserve fund.’^37^ Obesity was recognised by doctors acting for life assurance companies as ‘an indication of imperfect health’, and disproportionately heavy individuals were either ‘“loaded” or declined as second- or third-class lives’ on the grounds that ‘[o]bese persons bear accidents badly, are unsatisfactory subjects for surgical operations, and are apt to succumb to serious illnesses’.^38^

Simultaneously, the new maternal and child health clinics which sprang up in France, Britain and the United States in the late nineteenth and early twentieth century swiftly established similar procedures for assessing the health of infants and young children. The systems instituted to bring mothers and infants under medical and state surveillance provided ample means to gather and collate measurements establishing ‘normal’ height/weight ratios throughout child development.^39^ As Lyubov Gurjeva has demonstrated, during the late nineteenth century middle-class households consolidated a model of ‘scientific’ childcare, informed by and incorporating the resources of childcare handbooks, advertisements on infant feeding (themselves advice rich and incorporating charts to record growth), and the tools of anthropometric measurement, weighing scales, height and weight charts and baby diaries.^40^ For middle-class parents, childcare, she argues, ‘was never just consumption of whatever was produced outside the home, but a production in its own right’, by means of the infant foods, measuring instruments, child-rearing manuals and tabulated records that were utilised with great enthusiasm. Thus, by the end of the nineteenth century, the ideal of a quantified norm of healthy weight—at least for infants and children—was well established, both in professional circles and in many middle-class homes. However, its move into general medical practice and out of the domestic nursery would be more gradual and less comprehensive. Indeed some doctors expressed specific resistance to standardised classificationism of adults, which they claimed bore little relevance to individuals, including overweight individuals.^41^ Dr Wilhelm Ebstein, author of an influential book on corpulence and its treatment, was still dubious about the value of statistical material in 1890, given the variation in age, lifestyle and bodily structure of individual patients.^42^

## Moving towards Measurement? Weight and Weighing

Such resistance to purely quantified norms of weight notwithstanding, after the mid-nineteenth century, an increasing array of books focused more explicitly on the problem of British corpulency or ‘embonpoint’ and the management of diet and weight. William Banting (1796–1878), undertaker by profession and one of the earliest and best-known marketers of a ‘scientific’ diet programme, unambiguously rejected much of the advice offered by his physicians concerning the ‘naturalness’ of his hefty, ageing body. He adopted instead the low carbohydrate, carnivorous dietary system that made his name (in the process shedding 46lbs, at a rate of 2–3lbs every few weeks over the course of a year, and 12¼ inches round the waist).^43^ His best selling, *Letter on Corpulence*, first published in 1863, went into its twelfth edition by 1902.^44^ For Banting, it was not mere fat that caused his crisis and determination to slim, but obesity which caused him pain, inconvenience, ill health and ridicule.

Although no very great size or weight, still I could not stoop to tie my shoe, so to speak, nor attend to the little offices humanity requires without considerable pain and difficulty, which only the corpulent can understand; I have been compelled to go down stairs slowly backwards, to save the jar of increased weight upon the ancle [*sic*] and knee joint, and been obliged to puff and blow with every slight exertion . . .^45^

Although his work was not unprecedented, in terms of visibility and impact, Banting was widely regarded as a trailblazer. He detached dietary interventions from medical therapy and placed them firmly in the realm of self-help and self-monitoring. Moreover, after 1864 his book included a height–weight table, taken from Hutchinson’s insurance tables, to guide his readers on their targets for good weight and health. Specifically, he urged his followers ‘to get accurately weighed at starting upon a fresh system, and continue to do so weekly or monthly’. This act of quantification would not merely verify their progress, but ‘arm them with perfect confidence in the merit and ultimate success of the plan’.^46^

Importantly, whether advocating or deprecating medical guidance, most texts implicitly assumed or explicitly asserted that weight management would take place at home, where most meals were prepared and consumed, and most advocated weighing. Thus in 1850 Dr Thomas King Chambers, asserted in his study of corpulence, that ‘I am disposed, then, to think we cannot have a better test of the increase of fat than in the indications afforded by the balance.’^47^ Yet even having asserted the utility of Hutchinson’s tables, his support of measurement was not unqualified; it was, he claimed ‘impossible . . . to fix any absolute standard of weight’ and ‘incorrect’ to use such ‘average’ height/weight ratios as establishing individual healthy weights. These caveats notwithstanding, he acknowledged that the transgression of ‘certain limits on each side of the average’ could predispose individuals to, or even become, ‘infirmity’. Dr A. W. Moore’s (1853–1909) *Corpulency; i.e. Fat, or, Embonpoint, in Excess . . . Explaining Briefly his Newly-Discovered DIET SYSTEM* associated fatness largely with constitution and predisposition rather than disease, and emphasised self-management in preference to the intervention of physicians. His small volume included an innovative ‘Diet Diary’, laid out in columns for the reader to complete, detailing what they ate each day, which was also to be carefully measured and weighed. The table provided space for recording changes in individual weight (implying access to weighing apparatus of some kind) and instructed that dieters implement ‘a ruthlessly organised system of watchfulness and intent’.^48^ A *Morning Post* reviewer lauded Moore’s regime, with its emphasis on self-monitoring, for its simplicity and straightforward approach: ‘without any further medical aid than the pamphlet affords he can set to work to lessen the weight of his body. The plan of treatment is simple, and in its explanation devoid of all medical mystification.’^49^

In contrast, Dr Watson Bradshaw was keen to discourage ‘rash experiments’ in dieting (‘domestic medicine is fraught with innumerable evils’) and set out a detailed programme for the overweight in *On Corpulence*, published in 1864.^50^ However, here too, day-to-day management was envisaged as the responsibility of the patient, with the aim of steady reduction, ‘slow, safe, and certain measures’; through avoidance of alcohol and sugar, moderate food intake, exercise and keeping the stomach empty for as long as possible, ‘the result may almost be regarded with statistical certainty’.^51^ Nathaniel Edward Yorke-Davies’ *Foods for the Fat: A Treatise on Corpulency,* first published in 1889, urged that being overweight required the intervention of a physician, who would provide treatment, an individual diet plan, and crucially the necessary *moral* support to carry out a cure.^52^ In a piece published in the widely read news and general interest journal, *The Gentleman’s Magazine*—a mine of information on medical matters—he emphasised the serious risks of obesity; it strained the heart, stomach and lungs, and could reduce life expectancy.^53^ Despite these risks, by the time that the individual became entangled in the toils of corpulency, which crept on ‘insidiously and slowly’, Yorke-Davies argued, ‘he or she finds, when it becomes necessary to grapple with it, the power to do so curtailed, and the effort of taking the necessary steps so burdensome as to be practically impossible or too painful to continue’.^54^

Diet books stepped up their emphasis on science and nutrition towards the end of the century, and Yorke-Davies referred to the task of the dietician and chemist in shaping knowledge about rational food intake. Like other dietary guides, his book acknowledged better-known diet regimes, including Banting’s diet and Ebstein’s formula, but criticised the first for its extremity and the latter for its incorrect science and for recommending too large an intake of fat. While claiming to draw on scientific knowledge and approaches to fix the problem, and emphasising careful regulation by the physician, his diet plan relied largely on the straightforward correction of the diet and additional exercise. Yorke-Davies provided his readers with recipes and advice on food intake based on hospital dietaries and in relation to expenditure of effort in work and physical activity. Food was to be carefully weighed, and reports of successful weight loss indicated that his dieters, too, were regularly weighed recording their week-by-week weight reduction. His most famous diet patient was American President William Howard Taft (1857–1930), who hired York-Davies to supervise his weight loss programme. The two corresponded regularly for over 20 years and Taft kept a daily record of his weight, alongside details of his food intake and physical activity.^55^ Yorke-Davies’ book enjoyed enduring popularity, appearing in its 17th edition (35,000 copies) in 1906; aside from the patients who consulted him at his Harley Street practice, others lived abroad and treatment took place via correspondence.^56^

The inconsistency with which self-weighing was both advised to householders and practised by them reflects more than medical or domestic ambivalence about the technique. In fact, the acquisition of accurate and comprehensive measurements of height and weight for healthy adults proved a greater challenge than was the case for infants. Initially demanding complex systems of weights and the specific positioning of individuals under surveillance, early instruments for quantifying adult body weight made them unlikely domestic—or indeed clinical—technologies. Moreover, as the editors of the *Lancet* indicated in 1897, personal weighing scales had been too large (and too expensive) even for use in medical consulting rooms, much less domestic spaces, though new designs promised cheaper and more compact solutions.^57^ Unlike scales for infants, scales suitable for adults were at first cumbersome and prone to inaccuracy. Users required training expertise (and often a second pair of hands) to produce accurate measurements.^58^ Thus despite popular and professional recognition of weight as a valuable indicator of health, its systematic measurement was not fully embedded into general practice until the turn of the century, though scales were utlilised in institutions such as schools, prisons and by the army.^59^

Nonetheless, as early as 1883, the Lancashire and Cheshire Branch of the British Medical Association heard proposals from the BMA’s Collective Investigation of Disease Committee that members should make patient self-surveillance—especially of weight and growth—‘a new fashion in England’: ‘people should adopt a new system in the way of albums, and record the histories of their own lives; . . . they should be taught—and you doctors will have to teach them—*to watch themselves intelligently*’. They would likewise be taught to measure themselves and their children against standardised height and weight curves. Speaking for the Committee, Dr Frederick Akbar Mahomed celebrated ‘the extreme benefit of the weighing machine in medicine’. It was not only ‘the most useful thing that a medical man can possess’ but explicitly a tool that ‘the father of every family’ should have at home. ‘Nothing’, Mahomed claimed, ‘will so soon tell him when his children are wrong as a fall in their weight’.^60^ Mahomed extolled weight as a predictive diagnostic tool, citing reports of its use in the USA (though he refrained from recommending the weekly weighing advocated for young children in Boston). But not everyone in the audience was persuaded; indeed, one listener complained (albeit ‘jocularly’) ‘that the plan . . . will tend to make people rather too introspective, and that we shall have more *malades imaginaires* in the future than we have at the present time’.^61^

Working with Francis Galton, Mahomed produced the Albums according to his own specifications, again urging ‘every parent’ to purchase a home scale since ‘the accuracy of public weighing machines cannot always be depended upon’. The expense, he assured readers, would be recouped through ‘the increased facility’ home weighing offered in ‘managing the health of children’.^62^ Yet despite enthusiastic professional advocacy, neither the *Life History Album* nor self-weighing became ‘the fashion’, and the Album fell out of print.^63^ It is likely that the relatively high cost required to maintain the albums—not least, the expense of the regular weighing it required—put such ‘intelligent’ self-surveillance out of reach for most. An advertisement at the back priced a suitable ‘combine weighing and measuring machine’ at £5 5s, while several firms of surgical instrument makers charged sixpence each time to ‘weigh and measure’ individuals.^64^ In any case, advertisements in the *British Medical Journal* illustrate that adult ‘personal weighing machines’ were still marketed as novel articles of consulting room furniture in 1911.^65^ Even insurance companies only sporadically required the production of exact body weights from their clients before the 1920s, relying instead on height, chest and abdominal dimensions to generate estimated weights (and indeed to correct for inaccurate or fraudulent scale measurements).^66^

Even so, the convinced and the curious alike had long found ways and means to weigh. In his 1856 diet book, A. W. Moore favoured weighing machines ‘found at a railway station, or at any place where they are used for commercial purposes’. The very public positions they occupied ensured heavy people would become a ‘source of amusement’; ‘the lean ones on these occasions, admiring the beauty of their lankiness, smile with a kind of self-complacency at the heavy ones, whilst making them the butt of their jokes’.^67^ Lisa Coar has noted that Banting’s *Letter on Corpulence* intensified concern with correct weight and triggered a proliferation of weighing machines in public outlets, largely utilised by men.^68^ Observing this new fixation with dieting, a review in *Once-a-Week* despaired about the potential demise of jolly, rotund mayors and aldermen, ‘John Bull transformed into a scarecrow’. In one well known hairdressers, the article reported, ‘we were struck with the number of persons who were weighing themselves’. Indeed, customers were greeted by a young lady holding a card showing Dr Hutchinson’s height-weight scales.^69^ And for the affluent visitor to spa towns, public and proprietorial scales, in conjunction with personal quantified weight records, had acquired a central role by the late 1880s. One British visitor recorded with amused wonder, the weighing practices of ‘the fat men of Carlsbad’:

One of the chief institutions of Carlsbad is the weighing machine; nowhere do you see so many weighing machines. . . . To be weighed is part of the day’s ceremonial; a very precise invalid has himself weighed before breakfast and after dinner, and before going to bed at night. . . . His weight is registered solemnly in the proprietor’s ledger, and checked by his own private records in his pocket-book. . . . [E]ach past year’s record is compared with the present, so that at Carlsbad the fat man grows thin—and the thin man grows fat.^70^

Though he was unclear about where actual weighing should take place, F. Cecil Russell’s, *Corpulency and Cure*—a booklet promoting his weight-reducing vegetable preparation—insisted that the efficacy of his nostrum could be tested by ‘stepping on a weighing machine after 24 hours’.^71^ His clients’ testimonials were fulsome in describing their substantial weight loss and improved health and outlook. Many were clearly weighing themselves regularly and checking for weight loss and weight maintenance. Other clients, however, reported that it was not convenient to get weighed, and defined their success by other means, both quantifiable and experiential. One Eastborne lady declared ‘I have not attended to the weighing but I am reduced in the waist about four inches. My appearance and feelings tell me I am sufficiently reduced; but what I am most thankful for is feeling so much better. I have quite lost that dreadful feeling of oppression and weight . . . M.H.’^72^

As we have seen, the adult personal scale took neither the doctor’s surgery nor the home by storm in the nineteenth century. Bulky, expensive and difficult to use, it was initially a specialists’ tool. However, the powerful new, and increasingly compact, compound technology embodied by the platform scale and the standardised height/weight table effectively severed the ties between experiential clinical knowledge and the diagnosis of health risks, enabling a lay fusion of health promotion and hobbyism. By the first decades of the twentieth century, the growing accessibility of adult weighing scales and the means by which to interpret weight readings would enable British laymen and women—just like the insurance assessors and medical practitioners so assiduously targeted by the scale’s early advocates—to form their own opinions of their weight ‘without being dependent on the opinion of another’. And as Katherine Vester has demonstrated for American consumers, this is exactly what they did.^73^ Broadbased concern—moral as well as medical—with weight, appetite, dietary consumption and bodily dimensions created by 1900 a context in which many cultural cues promoted slimness.^74^ Ina Zweiniger-Bargielowska has observed that, positioned as ‘race mothers’ and patriotic empire builders respectively, women and men alike were increasingly duty-bound to cultivate a ‘normal’ body for the good of the nation.^75^ Moreover, the medical profession had already educated women in particular, as the guardians of family health, to appreciate the health benefits of precise, numerically quantified knowledge of their infants’ weight, while cookery books and ‘domestic science’ had inculcated habits of precise measurement in relation to food and diet. The advent of the personal scale and the height/weight table simply allowed men and women to apply to their own bodies the same level of surveillance and shaped a heightened fad for dieting.^76^

Moving the scales out of the public health clinic and the doctor’s consulting room and into the often cramped spaces of the middle-class home required innovations in equipment and manufacture that did not enter the mass marketplace until the 1910s. As we have seen, however, popular cultures of self-surveillance got a head-start from the 1885 emergence of the penny-scales as a form of entertainment and self-help. For adults, at least, self-measurement was often a public activity, appealing to both men and women. As early as 1910, humorous postcards depicting such scales were commonplace, and by 1911, the *Encyclopaedia Britannica* noted that an ‘automatic personal weighing machine’ was to be found ‘at most railway stations’.^77^ Its very public nature poised such popular weighing at the intersection of entertainment, surveillance and health promotion.^78^ A British railway platform scale, branded by its Glasgow manufacturer as the ‘Auto-way Barometer of Health’, jocularly urged waiting passengers to ‘give yourself a weigh’. Its large dial simultaneously ensured that they would ‘give themselves’—or at least their weights—‘away’ to any interested passers-by at the same time.^79^ In contrast, British manufacturer Avery took a more serious tone in sales literature aimed towards shop proprietors: ‘The modern chemist’s shop is not complete without an automatic weighing machine’, and ‘[t]he clean hygienic appearance makes it an added attraction to any establishment’.^80^ Photographs and accounts of Britain’s pre- and interwar high streets certainly reveal penny scales appearing outside chemists’ shops, demonstrating the growing association between bodily quantification and other practices of health preservation. Yet like Hutchinson’s spirometer half a century earlier, interpretive guides were often embedded in these technologies: users required—or at least were assumed to require—guidance in interpreting their weights.^81^ In 1928, Sir J. Fortescue Flannery, chairman of W. and T. Avery, Ltd (one of two major UK manufacturers of weighing equipment) proudly informed his shareholders at the Annual Meeting: ‘The doctors tell us that we should frequently record our weight in order to judge the state of our health, and there is now on the market a personal weighing machine of extraordinary refinement which, for a penny in the slot, will instantaneously record personal weight and issue a printed ticket.’^82^ In 1932, scales were even incorporated into a Nestle confectionery promotion, via a specially designed railway platform scale that dispensed a ‘full sized tablet of Nestle’s milk chocolate and your weight’ to customers.^83^ The ubiquity of such scales well into the post-war period indicates that they retained their attractiveness and utility both to shopkeepers seeking to increase footfall and to customers unable to weigh themselves at home.

## ‘Should be in every household’: Selling Health through the Bathroom Scale

As ideals of precision and quantification entered public, domestic and professional understandings of health, what became of the home as a site of health care, consumption and decision making in the twentieth century? For decades, historians of medicine have told a story about the rise of science and the rise of the medical profession. That story usually plots an upward curve of professional status and power against a downward trend in patient agency, slowing only with the rise of ‘patient activism’ in the 1970s and 1980s. Closer attention to practices within the household, however, may challenge this implied correspondence between the growing cultural capital of biomedicine, and increases in its reach and impact on the ground—or in the home. How do the processes by which householders navigated the growing superabundance of readily accessible medical advice and goods relate to ideas and expectations about the home and its occupants in medical ‘modernity’? Drawing on the example of the bathroom scale in Britain, the remainder of this paper will explore the twentieth-century rise of quantitative, technologised self-surveillance as a domestic, home-based culture of health promotion, and an established health-seeking behaviour.

Even after the invention of the (relatively) small spring-balance platform scale in the 1910s, the purchase of a domestic adult scale represented a significant investment in self-surveillance even for a middle-class household. Its cost notwithstanding, as early as 1901 the journal *Womanhood* urged the overweight ‘patient’ to weigh herself regularly every fortnight to ascertain the progress of weight loss.^84^ The same journal also commented on the difficulties of ‘irksome’ self-monitoring at home. Nonetheless it became a preoccupation of the journal’s readership, which in 1903 produced its own height and weight table to guide its readers. Ada Ballin, founder of the journal (aimed at the ‘New Woman’, catering for the intellectual and physical needs of educated women), reported her own weight-loss efforts, indicating the permeation of weight quantification amongst her readership. Feeling compelled to lose a stone and a half in weight, she developed a robust regime of cold baths, beating and kneading ‘the fat bits’, exercise and sport, a modest diet and regularly took Vichy or Kisssengen Salts (advertised in the magazine as an aid to digestion).^85^ A 1911 British medical supply catalogue advertised an adult home scale for 22 s 6d—over £90 in current currency, and in relation to the incomes of the day, comparable in cost to the purchase of a new flat-screen television for consumers in 2012.^86^ Reflecting the likely purchaser, its copy portrayed a prosperous and robust man, dressed in ornately frogged pyjamas and leather slippers, standing precariously on the then-state-of-the-art ‘portable personal weighing machine: automatic action, guaranteed accurate’. These scales appeared alongside an array of precision measuring equipment, from dispensing scales to graduated infant feeding bottles. While much of this equipment was clearly intended principally for medical or nursing professionals and establishments, the catalogue stated firmly that the ‘personal’ scale ‘should be in every household’.^87^

By the 1920s, personal scales were well established as useful aids in monitoring weight loss and gain, reinforcing self-surveillance, responsible healthful behaviour and holding back the ageing process. Margaret Hallam explained that ‘“too, too solid flesh” is a burden to everybody’ in her 1921 manual *Health and Beauty for Women and Girls*.^88^ She suggested alongside the extensive exercise regime promoted in her physical culture manual: ‘The ideal thing . . . is to exercise extreme watchfulness, and never let the figure go’. Having ‘ascertained what the *normal* weight of the body should be . . . try to keep to within a few pounds of that weight either way’; she advised weighing every month on the same machine.^89^ The domestic weighing scale was also incorporated into rituals of weight loss involving the use of slimming products and bathing techniques, such as Clark’s Thinning Bath Salts, to refine ‘the Too Stout Figure Into Lines of Slender Beauty’. A scantily draped young woman was depicted weighing herself on large domestic scales next to her bath, accompanied by the promise of ‘harmless and healthy reduction of size and weight, after every bath, that you can see shown on the weighing scales’.^90^ Here, the scales figured both as proof of efficacy and reward for persistence. A chapter on slimming in *The Modern Woman*, entitled ‘Cheating the Scales’, indicated their regular usage as well as the potential to assert superiority over the mechanical weighing apparatus through careful dieting.^91^

The popular press swiftly incorporated the personal scale into its prescriptive canon of regimen and self-control. Bringing the latest scientific studies of physiology and dietetics to their readers in 1916, the British *Nash’s and Pall Mall Gazette* reminded its audience, presumed to be upper-middle-class men with sedentary occupations, that they might ‘readily regulate’ their diets and control their weight. They needed only to weigh each item of food and consult the newly available caloric tables, keeping in mind that the normal body required only ‘sixteen calories per pound of body weight . . . every twenty-four hours when you are not exercising’. The article, devoted to expounding the virtues of lower protein consumption for the human ‘machine’, presumed easy access to both kitchen and bathroom scales. For the man unwilling to commit to such a meticulous regimen for metabolic efficiency, it advised moderation and self-control at the table and to ‘check results now and again, weighing yourself to see whether you are gaining or losing weight, modifying your fuel intake in accordance with the record of the scales’. If men would not take ‘at least that much trouble in the proper fuelling’ of their ‘bodily machine’, they ‘should not complain of ill-health’ or foreshortened lives.^92^

The greater seriousness with which private acts of self-help and surveillance, including private self-weighing, were imbued is also evident in the ways in which the ‘personal weighing machine’ was marketed in Britain. While UK advertising, like that of US manufacturers, stressed that ‘[w]eighing is now a daily practice’, the private bathroom scale in Britain was not represented in the same glamorous light. In the early 1930s, advertisements might combine images of kitchen balances (‘why not a practical Christmas present this year?’) with representations of the massive public scales that still served as ‘personal weighers’ for most Britons—and would do until well after the Second World War.^93^ Even later in the decade, bathroom scales were marketed ‘for all the family’, rather than for individual adults.^94^ By 1936, Salter advertised its ‘personal weigher’ (now a small, flat bathroom model) not as an adjunct to beauty, but as a tool of hygienic citizenship: ‘These arduous days’, it admonished, ‘it’s a national duty to keep fit. Check your weight daily . . .’. The scale was explicitly described as a way for family members to ‘check their health’ and to preserve it for the nation.^95^

**Fig. 1:**
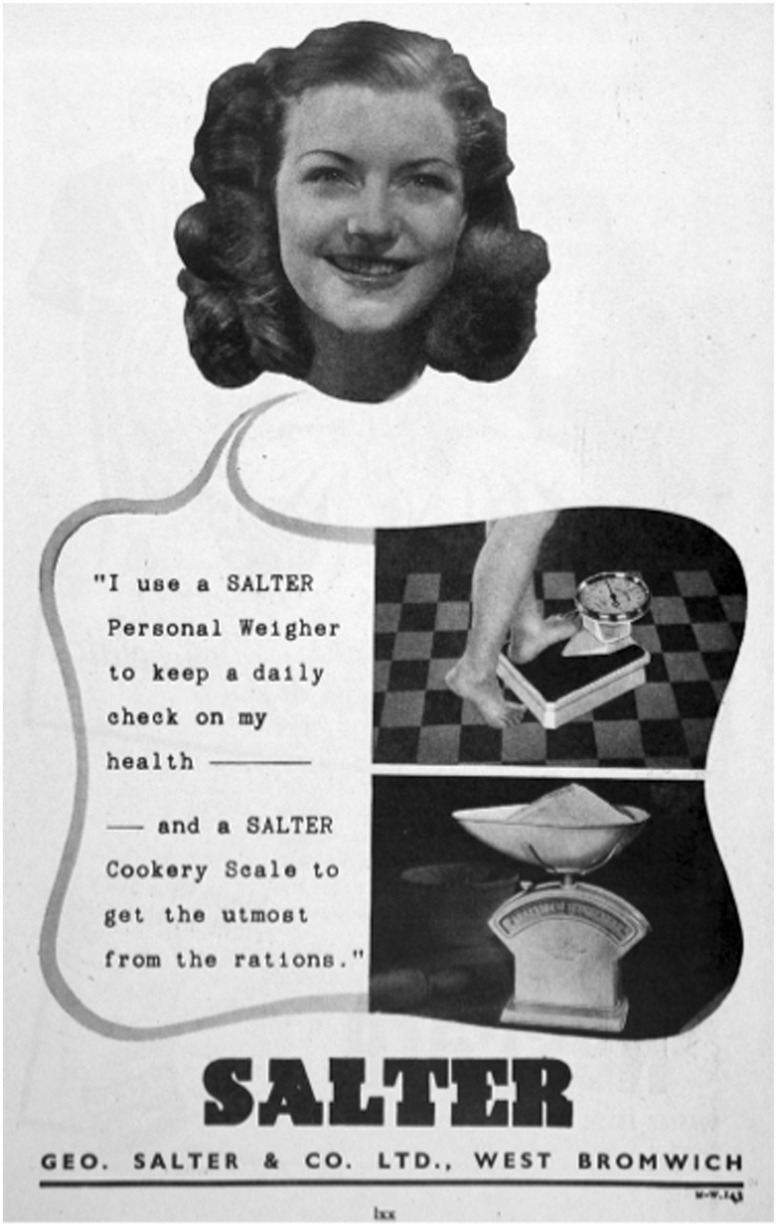
Geo. Salter & Co. Ltd., ‘I use a Salter . . .’ 1947, various publications <http://www.gracesguide.co.uk/File:Im1947MHI-Salter.jpg> accessed 31 July 2013. Courtesy, Grace's Guide to British Industrial History.

**Fig. 2:**
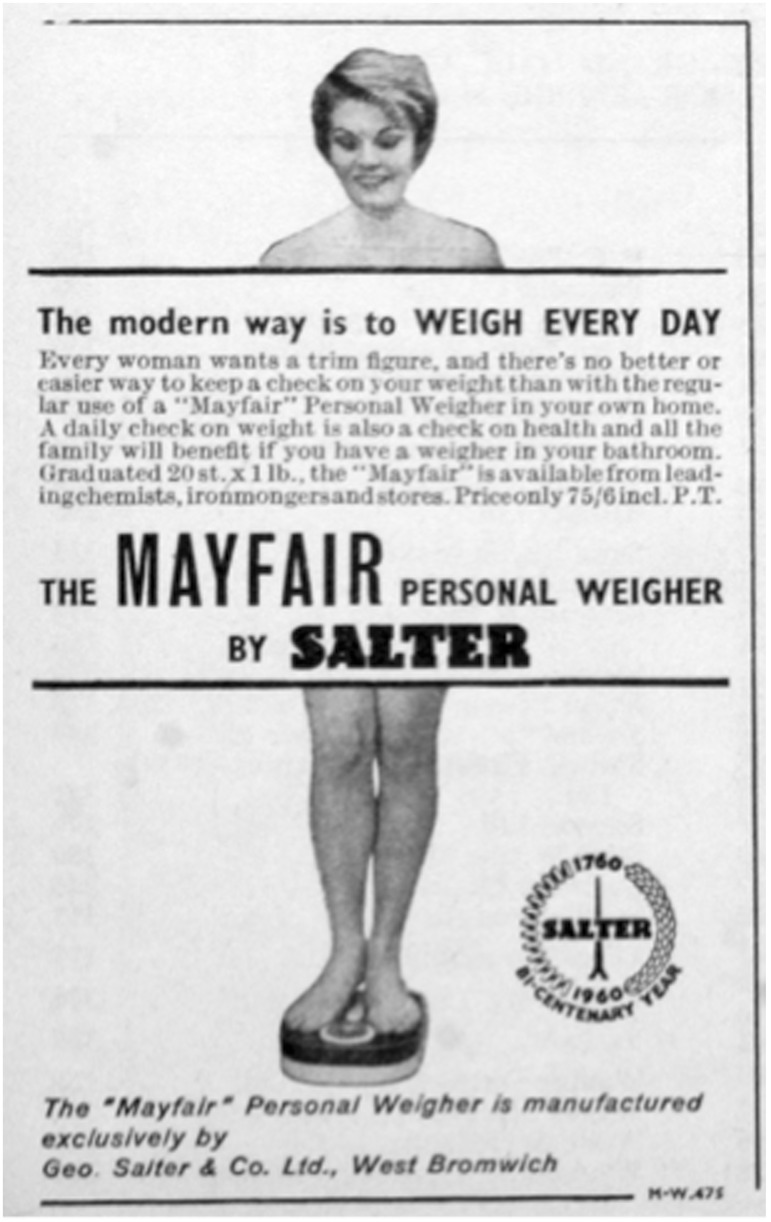
Geo. Salter & Co. Ltd., ‘The Modern Way is to Weigh Every Day’, 1960, various publications, <http://www.gracesguide.co.uk/File:Im196003IHX-Salter.jpg> accessed 31 July 2013. Courtesy, Grace's Guide to British Industrial History.

This focus on health, utility and the combination of kitchen and personal scales would continue through the war years and beyond. Even in 1947, when US scale makers had enthusiastically embraced the pursuit of beauty, Salter continued to focus on the scales as a tool ‘to keep a daily check on my health’ and ‘to get the utmost from the rations’^96^ (Figure 1). Only in 1960 would the aesthetic motive explicitly enter Salter’s advertising, and, even here, it was firmly linked to national modernity. While the imagery—a woman’s smiling face and apparently bare torso and legs peeked out from behind the ad’s text—echoed US copy, the strapline claimed ‘[t]he modern way is to weigh every day’, and linked the quest for a ‘trim figure’ back to the pursuit of health for ‘all the family’. At £3 15 s 6d (£67.80 in 2010) the scale was beyond the reach of many working-class families (doubtless contributing to the continued popularity of the public scale), but only the scale’s ‘Mayfair’ branding hinted at the association with luxury and self-indulgence that remained so firmly a part of US advertising^97^ (Figure 2).

Even as personal scales themselves became smaller and more suited to domestic use, the idea that self-weighing was potentially a public and even a competitive activity persisted, at least in Britain. In 1952, two female visitors (G. and M.) to the Isle of Man sent Lancashire friends a typical saucy postcard. On the front, a scrawny, kilted man shared a railway platform scale with his buxom female companion under the text ‘Now Maggie, what’s half of 19 stone 3½ pounds?’ On the back, G. reported that they too had ‘all got weighed’, and regretted that she hadn’t thought of a similar scale-sharing scheme in time: ‘I should have thought of *this* idea. [M.] is thrilled to bits because I am now 7 pounds heavier than her’.^98^ A 1956 *Times* leader even celebrated the persistence of the ‘old English custom’ of ‘getting weighed’:

[t]he number of weighing machines on our piers and promenades and railway platforms, in chemists’ shops and fun-fairs and snack bars is as large as, if not larger than ever it was. . . . There is always some small boy or stout middle aged gentleman or plump or pining maiden regarding that quivering pointer with glee or anxiety or complacency. . . . When customs are truly rooted in the hearts and lives of the people they need no subsidy for their survival.^99^

The editors ascribed the public scale’s enduring popularity not only to habit but to childhood training: ‘The child who in the first months of life is cradled on the scale, whose every ounce is charted with loving care, is father to the man who waiting on a train on any platform anywhere cannot resist the lure of the weighing machine.’ Unlike its serious domestic counterpart, public weighing was also cast as an act of harmless self-indulgence. The scales were a ‘temptation’, but an affordable and even a profitable one; ‘It is cheap . . . if it tells the slimming maiden that she has lost an ounce, she will not grudge the coin and if it warns the corpulent merchant that he has put on another pound or two, well he is getting more for his money.’ Self-weighing, the editors concluded, was universally appealing because it was ‘always about that subject of inexhaustible interest—us’.^100^

By the 1950s, doctors too strongly advocated the use of domestic weighing scales, and urged their regular use to monitor weight gain and as ‘instruments of prevention’. An article in *Family Doctor*, published by the BMA for household consumption, stressed that dieting ‘demands regular use of the scales, preferably in the bathroom where we can judge ourselves naked, having first consulted a table of weights and heights and set ourselves a standard for our age’.^101^ Another piece in the same publication described how 5 million consultations with doctors were with ‘fat people’ at risk of heart disease, high blood pressure, diabetes, strain on other organs, varicose veins, etc. Implicit in this narrative was a new message of national duty, this time to protect Britain’s new (but already stretched) National Health Service from avoidable cost.

Bathroom scales are a sound investment for health. It is important to weigh yourself regularly. It is much easier to check weight gains early than to slim after you have become overweight.

Dr Hutchin, author of the article, urged the use of bathroom scales as they enabled true weight to be established, without clothing, and for weighing to occur at the same time of the day with the scales in the same position.^102^

Nonetheless, private weighing remained linked to the middle-class demographic of British scale ownership, and the bathroom scale remained a rarity even within this group. Writing in the *Lancet*’s long-running ‘In England Now’ column another medical correspondent pondered the furtive use of his own bathroom scale by his would-be self-surveillant guests. Kept in the (fashionably upstairs) bathroom, his ‘weighing-machine’ was ‘the most irresistible fitting for the attention of our guests’. Guests, he noted with amusement, made ‘the most unconvincing excuses for slipping upstairs to weigh themselves’. Privacy—about both the practice and the results of weighing—was at the heart of the bathroom scale’s appeal. Despite manufacturer and medical invocations of the duty to weigh for health, the act itself was ‘a deadly secret’. Yet, of course, in a poorly soundproofed and small British home, the privacy self-weighers sought was largely illusory. Vividly, the author described the sounds of the inevitable struggle, if not with weight, then at least with weighing: ‘we keep the weighing machine tucked under the wash-basin. It has to be pulled clear for anyone to stand on its little platform: the job cannot be done without one loud metallic clank for the outward trip, and another one to hide the evidence.’ Why could ‘these women’ not weigh publicly, or at least ‘accept the truth more cheerfully’? The author offered no explanation, but he does reveal how high the stakes had become: the scale’s pointer (‘the horrid thing’) served as ‘the proper mark’ of ‘their conception of ideal womanhood’. His narrative suggested that women were not alone in investing the scale with the power to define their self-perceptions. ‘At the weigh-in last night, I registered 188 lb., the same as [professional heavyweight boxer] Floyd Patterson. Mind you the weight distribution looks a bit different, with a much lower centre of gravity’, he began—before hinting that he too might have ‘jumped up and down’ on the scale ‘to be a fair match’ for the boxer.^103^

In 1968 Salter finally moved to capitalise on demand for a cheaper domestic bathroom scale, bringing out the ‘Slimway’ scale. At the same time, the company replaced its rhetoric of health with the language of ‘modern styling and slim appearance’ (in this case, of the scale rather than its users). Here, too, for the first time the advertising copy promoted affordability.^104^ As the bathroom itself came in from the cold, and became an established feature of most British families’ lives, so too did the bathroom scale.

## Conclusions

As the preceding discussion has illustrated, a multiplicity of providers and would-be advisors bombarded the household with suggestions, admonitions and an ever-expanding array of products and services all intended to moderate weight, produce health and encourage practices of self-monitoring. Evidence of the responses of individuals and households to this increasing range of advice on offer on weight loss, as well as detail about their interest in and ability to purchase diet aids, however, is patchy. We cannot be sure how far professional advice was taken up or whether householders proceeded to chart their own course towards weight loss, adapting or overriding the doctor’s guidance. Further research drawing on oral history might provide additional insight into approaches to diet and the balance between professional guidance and self-determination. However, the evidence we have found thus far suggests that, as consumers, but also as agents in their own right, individuals and families sifted the wealth of options to produce their own idioms of care and cure, their own regimens of healthy living. While medical professionals of all stripes perceived and expressed themselves as increasingly knowledgeable, powerful and authoritative, and as obesity increasingly came to be described as a condition requiring expert management given the risks to health and longevity, householders were by no means passively compliant. Some adopted dietary and exercise regimes or alternative systems of health maintenance to preserve themselves in good health and weight; others adopted and adapted the advice of physicians, though often in their own homes and under their own daily self-surveillance—and some, perhaps, just jumped up and down on the scales. Either way, individuals and families charted their own paths to well-being and weight maintenance.

By the late 1960s and early 1970s, the bathroom scale—and the culture of domestic self-weighing—was increasingly at home in Britain (as it had earlier become in the United States). In part this reflects changes both in household architecture and in weighing technologies. Both the scales and the self-monitoring they allowed, once proudly modern, invisibly melted into household routine over the course of the twentieth century. But does this illustrate the domestication of medicine, or the medicalisation of the home? Here medical reactions to the ‘personal weighing machine’ offer some insight. While the thriving manufacture of personal and public scales indicates that health-seekers and dieters actively sought out the new devices, the professional response was mixed. As we have illustrated, many practitioners incorporated precision weighing into health regimen advice, but expected—at least initially—to manage the quantification of adult body weight themselves. Guides to weight reduction produced in the interwar years stressed the need to consult a doctor (particularly if embarking on a strict, absolute minimum diet aimed at rapid weight loss), but also regular weighing at home and emphasised that success was ‘to be found in the willingness of the subject to submit himself to a disciplined diet’.^105^ In *Slimming for the Million* Dr Eustace Chesser targeted patients and physicians and encouraged serious self-reflection on the part of the overweight: ‘Take a good look at yourself! Weigh yourself up! For your shape and weight are very closely connected both with your bodily health and your outlook on life.’^106^

Yet from the 1950s onwards, as the social prevalence, medical significance and political prominence of obesity grew, some doctors sought to reclaim their leading role in weight management and reduction. Perhaps unconsciously echoing his early twentieth-century peers’ distaste for fashionable ‘slimming’, in 1959, Alvan Feinstein (now celebrated as the father of clinical epidemiology) complained that ‘many attempts at weight reduction are based on aesthetic, cosmetic, or psychological factors rather than by physiologic concepts or the statistical features upon which the tables are based’. He insisted that ‘obesity cannot be defined in a strictly numerical fashion’, but could only be diagnosed by the expert medical use of clinical ‘inspection and palpation’.^107^ Instead, dieting practices took yet another turn, becoming associated with self-help and mutual aid, and collective dieting and weighing coordinated by Weight Watchers and similar organisations, and seemingly slipped yet further from direct medical control.^108^ By the 1970s, some researchers insisted that while ‘increasing the regularity and extent of self-monitoring should facilitate the self-control process’, in fact ‘daily weighing under . . . a slow rate of reduction would result in reduced motivation and participation’.^109^ They advised patients to be weighed only by study staff.

At the same time, many researchers have attacked the numerical relationships between body weight and height on which self-surveillance had been based since its inception: height/weight tables and the Body Mass Index have been regularly (and rightly) condemned as imprecise and unable to account for human variability in bone structure, muscle mass and fat distribution. Instead, specialists have advocated newer, more expensive, and importantly for our purposes, consumer-inaccessible technologies ranging from ‘novel handheld devices’ to ‘dual-energy X-ray absorptiometry’.^110^ Yet the NHS has rejected such high-tech tools in favour of adding to the simple tape measure to the bathroom scales already so thoroughly ensconced in the domestic health repertoire.^111^ While specialists may seek to influence a politically sensitive and commercially expanding field, the state is eager to enlist the public in their battle against the ‘obesity epidemic’. Weight, its surveillance, and its control are, it seems in the home to stay—demonstrating not only the durability of the bathroom scale, but of moral, rather than exclusively medical systems, for managing embodiment.

## Footnotes

^1^See, for example, John Harley Warner, *The Therapeutic Perspective: Medical Practice, Knowledge, and Identity in America* (Princeton, NJ: Princeton University Press, 1997); Stanley J. Reiser, *Medicine and the Reign of Technology* (Cambridge: Cambridge University Press, 1978); Deborah Brunton, ed., *Health, Disease and Society in Europe 1800–1930* (Manchester: Manchester University Press, 2004), including chapters on the changing role of hospitals, the modern medical profession and access to health care, and Mark Jackson, ed., *The Oxford Handbook of the History of Medicine* (Oxford: Oxford University Press, 2014). Most famously, of course, Foucault argued that the emergence of morbid anatomy and the impersonally classificatory gaze vastly increased the authority of the clinician over the patient. Michel Foucault, *The Birth of the Clinic: An Archaeology of Medical Perception*, trans. A. M. S. Smith (London: Routledge, 1973), 107–48.

^2^The US literature, often responsive to America’s current ‘obesity crisis’, includes: Hillel Schwartz, *Never Satisfied: A Cultural History of Diets, Fantasies, and Fat* (New York: Free Press, 1986); Peter N. Stearns, *Fat History: Bodies and Beauty in the Modern West* (New York: New York University Press, 1997); R. Marie Griffith, *Born Again Bodies: Flesh and Spirit in American Christianity* (Berkeley, Los Angeles and London: University of California Press, 2004); Kerry Segrave, *Obesity in America, 1850–1939: A Social History of Social Attitudes and Treatment* (Jefferson, NC: McFarland, 2008); Deborah I. Levine, ‘Managing American Bodies: Diet, Nutrition, and Obesity in America, 1840–1920’ (unpublished PhD dissertation, Harvard University, 2008); Amy Erdman Farrell, *Fat Shame: Stigma and the Fat Body in American Culture* (New York and London: New York University Press, 2011); Charlotte Biltekoff, *Eating Right in America: The Cultural Politics of Food and Health* (Durham, NC: Duke University Press, 2013). For a study drawing largely on French examples, see George Vigarello, *The Metamorphoses of Fat: A History of Obesity* (New York: Columbia University Press, 2013), translated from French by C. Jon Delogu.

^3^George Cheyne, *The English Malady; or, a Treatise of Nervous Diseases of All Kinds* (London: Srahan in Cornhill, 1733). See also Anita Guerrini, *Obesity and Depression in the Enlightenment: The Life and Times of George Cheyne* (Norman, OK: University of Oklahoma Press, 2000); Lucia Dacome, ‘Useless and Pernicious Matter: Corpulence in Eighteenth-Century England’, in Christopher E. Forth and Ana Carden-Coyne, eds, *Cultures of the Abdomen: Diet, Digestion, and Fat in the Modern World* (New York: Palgrave Macmillan, 2005), 185–204; Roy Porter, ‘Laymen, Doctors and Medical Knowledge in the Eighteenth Century: The Evidence of the *Gentleman’s Magazine’*, in Porter, ed., *Patients and Practitioners: Lay Perceptions of Medicine in Pre-Industrial Society* (Cambridge: Cambridge University Press, 1985), 138–68.

^4^Lisa Coar has asserted that preoccupation with fat and the desire for slenderness was largely a male concern in nineteenth-century Britain: ‘“Abandon fat all ye who enter here”: (Dis)ordering the Male Body, c.1800–1910’ (unpublished PhD thesis, University of Leicester, 2014).

^5^Sander L. Gilman, *Obesity: The Biography* (Oxford: Oxford University Press, 2010), 3.

^6^Jean Anthelme Brillat-Savarin, *The Physiology of Taste: Or Meditations on*
*Transcendental Gastronomy*, trans. M. F. K. Fisher (first published in French in 1826; Washington, D.C.: Counterpoint, 1999), 245. Cited in Gilman, *Obesity*, 559.

^7^Louise Foxcroft, *Calories and Corsets: A History of Dieting over 2,000 Years* (London: Profile, 2013), 63; Takahiro Ueyama, *Health in the Marketplace: Professionalism, Therapeutic Desires, and Medical Commodification in Late-Victorian London* (Palo Alto, CA: The Society for the Promotion of Science and Scholarship, 2010), 81.

^8^Peter N. Stearns, ‘Fat in America’, in Forth and Carden-Coyne, eds, *Cultures of the Abdomen*, 239–57, 243.

^9^Hilary Marland, ‘“The Diffusion of Useful Information”: Household Practice, Domestic Medical Guides and Medical Pluralism in Nineteenth-Century Britain’, in Robert Jütte, ed., *Medical Pluralism: Past**—**Present**—**Future, Yearbook Medizin, Gesellschaft und Geschichte*, 2013, 46, 81–100, 85.

^10^Christopher J. Lawrence, ‘William Buchan: Medicine Laid Open’, *Medical History*, 1975, 19, 20–35; Richard B. Sher, ‘William Buchan’s *Domestic Medicine*: Laying Book History Open’, in Peter Isaac and Barry McKay, eds, *The Human Face of the Book Trade* (Winchester: St Paul’s Bibliographies, 1999), 45–64, 45.

^11^These included Thomas J. Graham, *Modern Domestic Medicine*, 2nd edn (London: Simpkin and Marshall, 1827); Graham, *Sure Methods of Improving Health, and Prolonging Life **… by Regulating the Diet and Regimen* (London: R.M. Timms, 1831); Jabez Hogg, *The Domestic Medical and Surgical Guide, for the Nursery, the Cottage, and the Bush* (London: Ingram, Cooke, and Co., 1853); J. H. Walsh, *Domestic Medicine and Surgery: With a Glossary of the Terms used Therein*, new and revised edn (London: Frederick Warne, 1875); *Gardner’s Household Medicine and Sick-Room Guide: A Description of the Means of Preserving Health and the Treatment of Diseases, Injuries, and Emergencies*, 13th edn (London: Smith, Elder, & Co.,1898). Information on diet was also included in guides on domestic economy, such as *The Practical Housewife: A Complete Encylopaedia of Domestic Economy and Family Medical Guide* (London: Houston and Wright, 1860), and on vegetarianism and its health benefits, see Anna Kingsford, *The Perfect Way in Diet: A Treatise Advocating a Return to a Natural and Ancient Food of our Race* (London: Kegan Paul, Trench, Trüber & Co., 1898); Alfred B. Olsen and M. Ellsworth Olsen, eds, *The School of Health: A Guide to Health in the Home* (London: International Tract Society, 1906).

^12^Charles E. Rosenberg, ‘Health in the Home: A Tradition of Print and Practice’, in Rosenberg, ed., *Right Living: An Anglo-American Tradition of Self-Help Medicine and Hygiene* (Baltimore and London: Johns Hopkins University Press, 2003), 1–20, 2.

^13^Marland, ‘“The Diffusion of Useful Information”’.

^14^For the production of advice and goods to facilitate the adaptation of ‘preventive therapy’ by tuberculosis sufferers to domestic spaces, see Graham Mooney, ‘The Material Consumptive: Domesticating the Tuberculosis Patient in Edwardian England’, *Journal of Historical Geography*, 2013, 42, 152–66.

^15^Martha H. Verbrugge, *Able-Bodied Womanhood: Personal Health and Social Change in Nineteenth-Century Boston* (New York and Oxford: Oxford University Press, 1988), 36.

^16^Graham, *Modern Domestic Medicine*, 158, 160.

^17^John Flint South, *Household Surgery: Or, Hints on Emergencies*, 4th edn (London: G. Cox, 1852), 334.

^18^*Gardner’s Household Medicine and Sick-Room Guide*, 57.

^19^J. M’Gregor-Robertson, *The Household Physician: A Family Guide to the Preservation of Health and to the Domestic Treatment of Ailments and Disease, with Chapters on Food and Drugs, and First Aid in Accidents and Injuries* (London, Glasgow, Edinburgh and Dublin: Blackie & Son, 1890).

^20^Olsen and Olsen, eds, *The School of Health*, 126.

^21^*Ibid*. See Margaret Barnett, ‘Fletcherism: The Chew-chew Fad of the Edwardian Era’, in David F. Smith, ed., *Nutrition in Britain: Science, Scientists and Politics in the Twentieth Century* (London and New York: Routledge, 1997), 6–28.

^22^This was part and parcel of broader efforts to encourage girls to manage their own health; see Hilary Marland, *Health and Girlhood in Britain, 1874–1920* (Houndmills: Palgrave Macmillan, 2013).

^23^Anna Bonus Kingsford, *Health, Beauty and the Toilet: Letters to Ladies from a Lady Doctor* (London and New York: Frederick Warne, 1886), 10.

^24^Emma D. Walker, *Beauty Through Hygiene: Common-sense Ways to Health for Girls* (London: Hutchinson, 1905), 80.

^25^E.g. M’Gregor-Robertson, *The Household Physician*.

^26^E.g. John Savory, *A Compendium of Domestic Medicine: and Companion to the Medicine Chest* (London: John Churchill, 1836), vii–xiii.

^27^Annemarie Jutel, ‘Doctor’s Orders: Diagnosis, Medical Authority and the Exploitation of the Fat Body’, in Jan Wright and Valerie Harwood, eds, *Biopolitics and the ‘Obesity Epidemic’: Governing Bodies* (New York and London: Routledge, 2009), 67; Vigarello, *The Metamorphoses of Fat*, 111.

^28^E.g. Stephen Jacyna, ‘Medicine in Transformation’, in William F. Bynum, Anne Hardy, Stephen Jacyna, Christopher Lawrence and E. M. Tansey, eds, *The Western Medical Tradition, 1800–2000* (Cambridge: Cambridge University Press, 2006), 7–101; John E. Lesch, *Science and Medicine in France: The Emergence of Experimental Physiology, 1790–1855* (Cambridge, MA: Harvard University Press, 1984); Russell C. Maulitz, *Morbid Appearances: The Anatomy of Pathology in the Early Nineteenth Century* (Cambridge: Cambridge University Press, 1987); John Pickstone, ‘Bureaucracy, Liberalism and the Body in Post-Revolutionary France: Bichat’s Physiology and the Paris School of Medicine’, *History of Science*, 1981, 19, 99–155.

^29^Adolphe Quetelet, ‘Sur la Taille Moyenne de l’Homme dans les Villes et dans les Campagnes, et sur l’Age ou la Croissance est Complètement Achevée’, *Annales d’Hygiène Publique*, 1830, 3, 24–6, quoted in Lawrence T. Weaver, ‘In the Balance: Weighing Babies and the Birth of the Infant Welfare Clinic,’ *Bulletin of the History of Medicine*, 2010, 84, 30–57. See also J. Rosser Matthews, *Quantification and the Quest for Medical Certainty* (Princeton, NJ: Princeton University Press, 1995); Jean-Guy Prévost and Jean-Pierre Beaud, *Statistics, Public Debate and the State, 1800–1945: A Social, Political and Intellectual History of Numbers* (London: Pickering and Chatto, 2012) 49–61, 49; Charles E. Rosenberg, ‘The Tyranny of Diagnosis: Specific Entities and Individual Experience’, *The Millbank Quarterly*, 2002, 80, 237–60; Gérard Jorland, Annick Opinel and George Weisz, eds, *Body Counts: Medical Quantification in Historical and Sociological Perspective* */ La quantification médicale, perspectives historiques et sociologiques* (Montreal and Kingston: McGill-Queen’s University Press, 2005).

^30^John Hutchinson, ‘On the Capacity of the Lungs, and on the Respiratory Functions’, *Medico-chirurgical Transactions*, 1846, 29, 137–252, 223. On John Hutchinson, see E. A. Spriggs, ‘John Hutchinson, the Inventor of the Spirometer: His North Country Background, Life in London, and Scientific Achievements’, *Medical History*, 1977, 21, 357–64.

^31^Hutchinson, ‘On the Capacity of the Lungs’, 223.

^32^Reiser*, Medicine and the Reign of Technology*, 94. See also Hughes Evans, ‘Losing Touch: The Controversy over the Introduction of Blood Pressure Instruments into Medicine’, *Technology and Culture*, 1993, 34, 784–807, 786–87; and on resistance, Richard H. Shryock, ‘The History of Quantification in Medical Science’, *Isis*, 1961, 52, 215–37, 223; Christopher Lawrence, ‘Incommunicable Knowledge: Science, Technology and the Clinical Art in Britain 1850–1914’, *Journal of Contemporary History*, 1985, 20, 503–20, 513–17.

^33^John Hutchinson, *The Spirometer, the Stethoscope, & Scale-Balance; Their Use in Discriminating Diseases of the Chest, and Their Value in Life Offices; With Remarks on the Selection of Lives for Life Assurance Companies* (London: John Churchill, 1852), 55.

^34^Spriggs, ‘John Hutchinson’, 359. On the use of his tables, see Amanda M. Czerniawski, ‘From Average to Ideal: The Evolution of the Height and Weight Table in the United States, 1836–1943’, *Social Science History*, 2007, 31, 273–96, 274.

^35^Hutchinson, ‘On the Capacity of the Lungs’, 160.

^36^Czerniawski, ‘From Average to Ideal’, 273–4, 286.

^37^‘The Standard of Weight and Height’, *Review of Reviews*, January 1909, 39, 229, 57.

^38^F. Parkes Weber, ‘Fatness, Overweight and Life Assurance’, *The Medical Magazine*, June 1901, 333.

^39^See Rima Apple, *Mothers and Medicine: A Social History of Infant Feeding, 1890–1950* (Madison, WI: University of Wisconsin Press, 2005).

^40^Lyubov G. Gurjeva, ‘Child Health, Commerce and Family Values: The Domestic Production of the Middle Class in Late-Nineteenth and Early-Twentieth Century Britain’, in Marijke Gijswijt-Hofstra and Hilary Marland, eds, *Cultures of Child Health in Britain and the Netherlands in the Twentieth Century* (Amsterdam and New York: Rodopi, 2003), 103–25.

^41^Jutel, ‘Doctor’s Orders’, 68.

^42^Wilhelm Ebstein, *Corpulence and its Treatment on Physiological Principles* (London: H. Grevel, 1890), 13.

^43^William Banting, *Letter on Corpulence, Addressed to the Public*, 3rd edn (London: Harrison, 1864), 28–9.

^44^*Ibid*., 1st edn (London: Harrison, 1863); Farrell, *Fat Shame*, 35; Foxcroft, *Calories and Corsets*, 73–5; Gliman, *Obesity*, 61–5.

^45^Banting, *Letter on Corpulence*, 1st edn, 10–11.

^46^Ibid., 3rd edn, 36, 43–4.

^47^Thomas King Chambers, *Corpulence; or, Excess of Fat in the Human Body* (London: Longman, Brown, Green, and Longmans, 1850), 62.

^48^A.W. Moore’s *Corpulency; i.e. Fat, or, Embonpoint, in Excess** . . . Explaining Briefly his Newly-Discovered DIET SYSTEM, to Reduce the Weight and Benefit the Health* (London: J. Sheppard, 1856); three editions by 1857; Foxcroft, *Calories and Corsets*, 65.

^49^*Morning Post*, 20 March 1857, 3.

^50^Dr Watson Bradshaw, *On Corpulence* (London: Philip & Son, 1864), 20; Foxcroft, *Calories and Corsets*, 72.

^51^Bradshaw, *On Corpulence*, 22.

^52^Nathaniel Edward Yorke-Davies, *Foods for the Fat: A Treatise on Corpulency* (London: Chatto & Windus, 1889).

^53^Nathaniel Edward Yorke-Davies, ‘Over-Stoutness: Its Discomforts and Dangers’, *The Gentleman’s Magazine*, May 1905, 298, 452–63, 456.

^54^Yorke-Davies, *Foods for the Fat*, v–vi.

^55^Deborah I. Levine, ‘Corpulence and Correspondence: President William H. Taft and the Medical Management of Obesity’, *Annals of Internal Medicine*, 15 October 2013, 159, 565–70.

^56^Nathaniel Edward Yorke-Davies, *Foods for the Fat: A Dietetic Cure of Obesity with Chapters on the Treatment of Gout by Diet* (London: Chatto & Windus, 1906); Levine, ‘Corpulence and Correspondence’, 565.

^57^‘Notes, Short Comments and Answers to Correspondents’, *Lancet*, 8 May 1897, 149, 1316; ‘A New Form of Weighing Machine’, *Lancet,* 2 April 1898, 151, 940.

^58^See e.g. Hutchinson, ‘On the Capacity of the Lungs’, 241.

^59^E.g. Percy Boulton, ‘Some Anthropometrical Observations, *British Medical Journal* (*BMJ*), 4 March 1876, 1, 280–2. Notably, Boulton cited Hutchinson’s work and concurred that ‘the weighing machine’ could be ‘a powerful agent in preventive medicine’, but added ‘adult weight is only of value in noticing variation from an ascertained standard’, in conjunction with height (p. 280). Instead he focused in meticulous detail on the weight of the fetus in utero, infancy and early childhood.

^60^‘Collective Investigation of Disease’, *BMJ*, 3 November 1883, 2, 1192, 891–2, 891. Emphasis added.

^61^‘Collective Investigation of Disease’, 892. On the Committee, and Mahomed’s collaboration with Francis Galton on the Life History Albums, see Alun D. Hughes, ‘Commentary: “On the Cards”: Collective Investigation of Disease and Medical Life Histories in the Nineteenth Century’, *International Journal of Epidemiology*, 2013, 42, 1–6. An album is digitised at <https://archive.org/details/lifehistoryalbu00galtgoog>, accessed 11 March 2016. See, for the BMA’s inquiries into collective investigation, Harry M. Marks, ‘“Until the Sun of Science … the true Apollo of Medicine has risen”: Collective Investigation in Britain and America, 1880–1910’, *Medical History*, 2006, 50, 147–60.

^62^Francis Galton, ed., *Life History Album Prepared by the Direction of the Collective Investigation Committee of the British Medical Association* (London: Macmillan, 1884), 3.

^63^Francis Galton, *Life History Album: Tables and Charts for Recording the Development of Body and Mind from Childhood Upwards, With Introductory Remarks*, 2nd edn (London: Macmillan 1902).

^64^Galton, ed, *Life History Album*, n.p. In another advertisement, well-established London wine and coffee merchants, Berry Bros, offered to weigh and record their own customers gratis, as they had done since 1765.

^65^‘Advertisment for Bailey’s Consulting Room Couches and Scales’, *BMJ*, February 1913, 1, 45.

^66^Czerniawski, ‘From Average to Ideal’, 277–81; Stearns, *Fat History*, 27; Schwartz, *Never Satisfied*, 89.

^67^Moore, *Corpulency*, 35.

^68^Coar, ‘“Abandon fat all ye who enter here”’, 41. Levine has also discussed how the scales became a form of public entertainment in the USA: Levine, ‘Managing American Bodies’, 82–3, 88–98; see also Schwartz, *Never Satisfied*, 186.

^69^‘Our Once Fat Friend’, *Once-a-Week*, 11 June 1864,10, 696–9, 696, 697.

^70^Ernest Hart, ‘Spray from the Carlsbad Sprudel. II’, *BMJ*, 26 November 1887, 2, 1176–8, 1177.

^71^F. Cecil Russell, *Corpulency and the Cure*, 18th edn (London: n.p., 1896), 36.

^72^*Ibid*., 57, 47.

^73^Hutchinson, ‘On the Capacity of the Lungs’, 160; Katherina Vester, ‘Regime Change: Gender, Class, and the Invention of Dieting in Post-Bellum America’, *Journal of Social History*, 2010, 44, 39–70.

^74^See e.g. Coar, ‘“Abandon fat all ye who enter here”’; Anna Silver Krugovoy, *Victorian Literature and the Anorexic Body* (Cambridge: Cambridge University Press, 2002); Pat Rogers, ‘Fat is a Fictional Issue: The Novel and the Rise of Weight-Watching’, in Elena Levy-Navarro, ed., *Historicizing Fat in Anglo-American Culture* (Columbus, OH: Ohio State University Press, 2010), 19–39.

^75^Ina Zweiniger-Bargielowska, *Managing the Body: Beauty, Health and Fitness in Britain, 1880–1939* (Oxford: Oxford University Press, 2010), 62–148.

^76^Vester, ‘Regime Change’, 41–5, 49–57.

^77^For cards, see e.g. Misch and Stock’s Addled Ads, ‘Wanted’ Series 93 (designed in England and printed in Prussia, *c.* 1910). See also Getty Images/ Hulton Archive <gty.im/3422985>, ‘1st July 1913: A man weighs his family while waiting for their train at Waterloo Station, London’ (Photo by Topical Press Agency/Getty Images, accessed 28 February 2016); W. Ay, ‘Weighing Machines’, *The Encyclopaedia Britannica: A Dictionary of Arts, Sciences, Literature and General Information*, 11th ed., 32 vols (New York: Encyclopaedia Britannica, 1910), vol. 28, 468–77, 471.

^78^On the US case, see Levine, ‘Managing American Bodies’, 82–4, 87–90, 97–101, where she links US enthusiasm for such public weighing with wider interests in anthropometry.

^79^View at <http://www.lassco.co.uk/?id=58&tx_evlasscoproductdetail_pi1%5Buid%5D=15740>, accessed 19 January 2015.

^80^See the South Wales Coal Fields Project at <http://www.agor.org.uk/cwm/themes/medicine_and_health/community_health/HealthandHousing.asp>, accessed 19 January 2015.

^81^See, for example, Getty Images/Hulton Archive <gty.im/3381019> ‘Circa 1930: A woman being weighed on a set of scales called “The Robot Doctor”’ (Photo by London Express/Getty Images, accessed 28 February 2016); Getty Images/Hulton Archive <gty.im/3426437> Harold Clements, ‘7th February 1949: Philip Edwards watching as his bride-to-be steps onto the scales’, accessed 28 February 2016.

^82^‘W. and T. Avery, Ltd. A Good Year’, *The Times*, 25 July 1928, 26.

^83^Geo. Salter & Co. Ltd., Nestle’s Chocolate Machine, 1933, viewable at <http://www.gracesguide.co.uk/File:Im1933GWRMus-Salter.jpg>, accessed 11 March 2016.

^84^Martyn Westcott, ‘Obesity’, *Womanhood*, 1901, 33, 185.

^85^Mrs Ada Ballin, ‘Obesity’, *Womanhood*, 1903, 11, 23–5.

^86^Garrold Surgical Sundries, 1911. Current costs estimated via the calculators at <http://www.measuringworth.com/ukcompare/>, accessed 31 July 2013.

^87^*Ibid*.

^88^Margaret Hallam, *Health and Beauty for Women and Girls* (London: C. Arthur Pearson, 1921), 84–5.

^89^Margaret Hallam, *Dear Daughter of Eve: A Compleat Book of Health and Beauty* (London: W. Collins, 1924), 185, 182.

^90^*Nash’s and Pall Mall Gazette*, October 1924, 74, 93.

^91^Lillian Bradstock and Jane Condon, *The Modern Woman: Beauty. Physical Culture. Hygiene* (London: Associated Newspapers [n.d. c.1936].

^92^Henry Smith Williams, ‘Eating to Live’, *Nash’s and Pall Mall Magazine*, August 1916, 57, 607–12. 612.

^93^Geo. Salter & Co. Ltd., ‘A Gift of Long-lasting Utility’, 1934, various publications, <http://www.gracesguide.co.uk/File:Im193412GHK-Salter.jpg>, accessed 31 July 2013.

^94^Geo. Salter & Co. Ltd., ‘Gifts that Appeal’, 1936, various publications, <http://www.gracesguide.co.uk/File:Im193601GHK-Salter.jpg>, accessed 31 July 2013.

^95^Geo. Salter & Co. Ltd., ‘These Arduous Days . . .’, 1936, various publications, <http://www.gracesguide.co.uk/File:Im19360906P-Salter.jpg>, accessed 31 July 2013.

^96^Geo. Salter & Co. Ltd., ‘I use a Salter . . .’ 1947, various publications, <http://www.gracesguide.co.uk/File:Im1947MHI-Salter.jpg>, accessed 31 July 2013.

^97^Geo. Salter & Co. Ltd., ‘The Modern Way is to Weigh Every Day’, 1960, various publications, <http://www.gracesguide.co.uk/File:Im196003IHX-Salter.jpg>, accessed 31 July 2013.

^98^‘Postcard to Mr and Mrs T.L.T., Lancs.’, 3 August 1952. Private collection.

^99^‘Old English Custom’, *The Times,* 16 August 1956, 7.

^100^*Ibid*., 7.

^101^Harvey Williams, OBE, MD, ‘Is Dieting Worth While?’, *Family Doctor*, February, 1962, 12, 94–5, 95.

^102^Dr Kenneth C. Hutchin, ‘Stop Killing Yourself with Kindness’, *Family Doctor*, December 1962, 12, 742–4, 742.

^103^‘In England Now’, *Lancet*, 30 December 1961, 278, 1450.

^104^Geo. Salter & Co. Ltd., ‘Slim the new way’, 1968, various publications, <http://www.gracesguide.co.uk/File:Im196802GHK-Salter.jpg>, accessed 31 July 2013.

^105^E.E. Claxton, *Weight Reduction: Diet and Dishes* (London: William Heinemann, 1937), vii, 57, 59.

^106^Dr Eustace Chesser, *Slimming for the Million: A New Treatment of Obesity. A Practical Guide for Patient and Physician* (London: Rich & Cowan, [1939]).

^107^Alvan R. Feinstein, ‘The Measurement of Success in Weight Reduction: An Analysis of Methods and a New Index’, *Journal of Chronic Diseases*, 1959, 10, 439–56.

^108^Jessica M. Parr, ‘Obesity and the Emergence of Mutual Aid Groups for Weight Loss in the Post-War United States’, *Social History of Medicine*, 2014, 27, 768–88.

^109^Alan Bellack, Ronald Rozensky and Jeffrey Schwartz, ‘A Comparison of Two Forms of Self Monitoring in a Behavioural Weight Reduction Program’, *Behavior Therapy*, 1974, 5, 523–30, 524.

^110^S. O. McDoniel, P. Wolskee and J. Shen, ‘Treating Obesity with a Novel Hand-held Device, Computer Software Program and Internet Technology in Primary Care’, *Patient Education and Counseling*, 2010, 79, 185–91; Nirav R. Shah and Eric Braverman, ‘Measuring Adiposity in Patients: The Utility of Body Mass Index (BMI), Percent Body Fat, and Leptin’, *PLoS ONE*, 2012, 7, e33308. doi:10.1371/journal.pone.0033308 <http://www.plosone.org/article/info%3Adoi%2F10.1371%2Fjournal.pone.0033308>, accessed 4 June 2013.

^111^<http://www.nhs.uk/Livewell/loseweight/Pages/Appleorpear.aspx>, accessed 22 December 2013.

